# Obesity as Inducer of Cognitive Function Decline via Dysbiosis of Gut Microbiota in Rats

**DOI:** 10.3390/brainsci14080807

**Published:** 2024-08-12

**Authors:** Hoda B. Mabrok, Asmaa A. Ramadan, Ibrahim M. Hamed, Doha A. Mohamed

**Affiliations:** Nutrition and Food Science Department, Food Industries and Nutrition Institute, National Research Centre, Dokki, Cairo 12622, Egypt; hoda.mabrok@gmail.com (H.B.M.); ae.ramadan@nrc.sci.eg (A.A.R.); hamed5858@yahoo.com (I.M.H.)

**Keywords:** cognitive function decline, high-fat diet, high-fat/high-sucrose diet, gut microbiota, 16S rRNA, metagenomics, obesity

## Abstract

Diet-induced obesity is a global phenomenon that affects the population worldwide with manifestations at both the phenotypic and genotypic levels. Cognitive function decline is a major global health challenge. The relation between obesity and cognitive function is a debatable issue. The main goal of the current research was to study the implications of obesity on cognitive function and gut microbiota diversity and its impact on plasma and brain metabolic parameters in rats. Obesity was induced in rats by feeding on a high-fat (HF) or a high-fat/high-sucrose (HFHS) diet. The results reveal that both the HF (0.683) and HFHS (0.688) diets were effective as obesity inducers, which was confirmed by a significant increase in the body mass index (BMI). Both diet groups showed dyslipidemia and elevation of oxidative stress, insulin resistance (IR), and inflammatory markers with alterations in liver and kidney functions. Obesity led to a reduction in cognitive function through a reduction in short-term memory by 23.8% and 30.7% in the rats fed HF and HFHS diets, respectively, and learning capacity and visuo-spatial memory reduced by 8.9 and 9.7 s in the rats fed an HF or HFHS diet, respectively. Bacteroidetes, Firmicutes, Proteobacteria, Fusobacteria, and Spirochaetes phyla were detected. The Firmicutes/Bacteroidetes ratio (F/B) significantly decreased in the HF group, while it increased in the HFHS group compared to the normal control. The two species, *Bacteroides acidifaciens* and *Bacteroides ovatus*, which are associated with IR, were drastically compromised by the high-fat/high-sucrose diet. Some species that have been linked to reduced inflammation showed a sharp decrease in the HFHS group, while *Prevotella copri*, which is linked to carbohydrate metabolism, was highly enriched. In conclusion: Obesity led to cognitive impairment through changes in short-term and visuo-spatial memory. A metagenomic analysis revealed alterations in the abundance of some microbial taxa associated with obesity, inflammation, and insulin resistance in the HF and HFHS groups.

## 1. Introduction

Metabolic syndrome is a chronic condition and a major risk factor for several comorbidities including obesity, steatohepatitis, diabetes mellitus, and cardiovascular disease [1,2]. Several triggering factors contribute to the development of metabolic syndrome including lack of physical activity and an atherogenic diet, which would consequently lead to IR. Long-term IR is considered the main causing factor of metabolic syndrome development and is widespread [3,4]. Driven by time, unhealthy food, and lifestyle patterns, metabolic syndrome could develop into a chronic condition of low-grade inflammation which manifests itself through different pathways involving diverse cascades of key metabolites and cytokines [5,6]. Obesity is a multifactorial chronic condition that has been closely linked to metabolic syndrome. Changes in diet, physical activity, and lifestyle habits can significantly affect the development and progress of obesity [7,8,9]. Globally, 39% of adults aged above 18 are overweight and 13% are considered obese with projected levels of 85–89% by 2030 [10,11]. In the Middle East, with obesity prevalence rates well above the global average, half the region’s adult women (50.1%) and more than two in five men (43.8%) are obese or overweight [12].

Different approaches have been adopted for managing obesity including behavioral intervention, pharmacotherapy, and nutrition [13]. Complementary medicine and nutraceuticals have been promising in treating the consequences of obesity in adults. Different probiotic strains have been used to control obesity either alone or with prebiotics. Among the most successful blends is the symbiosis between lactic acid bacteria and insoluble dietary fibers [14,15].

Obesity induced by a high-fat diet usually comes with implications on metabolic parameters not limited to lipid profile, blood glucose levels, and insulin resistance (IR) [16], which in turn plays an important role in the development of metabolic syndrome, type 2 diabetes, and cardiovascular disease [17]. The increasing demand for added-sugar foods and beverages worldwide is contributing to the problem of obesity and IR. A high sucrose intake, along with an increasing demand for processed foods and snacks, has spiked sugar consumption to unprecedented levels around the world [18,19]. Sucrose-sweetened beverages have been shown to elevate plasma triglycerides and promote inflammation [20,21,22]. In a previous National Health and Nutrition Examination Survey (NHANES) study, a clear association was established between abdominal obesity, high-sugar beverage intake, and higher levels of C-reactive protein (CRP) in study groups with and without prediabetes [23]. Growing evidence suggests that a high-fat/high-sugar diet may potentially increase oxidative stress, adipokines, and pro-inflammatory cytokines like TNF-α, INFγ, IL-6, and IL-1β [24,25].

The effect of metabolic disorders on intestinal ecology is becoming apparent with the latest nutritional and metabolomic studies [26,27,28]. Recent studies in nutritional genomics and metabolomics suggest a crosstalk between the gut microbiota and the biomarkers and metabolites involved in the incidence and development of metabolic syndrome and related chronic metabolic disorders including obesity, cardiovascular diseases (CVD), fatty liver, and type 2 diabetes [29,30]. Our previous work in this regard showed clear signs of gut microbiota perturbation and a loss of intestinal tight junction concurrently with the induction of steatohepatitis, diabetes, and related metabolic conditions in animal models [26,31,32]. The delicate balance between a state of homeostasis and dysbiosis can be easily disturbed by an unbalanced diet, high in dietary fat and sugar, leading to low-grade inflammation, increased intestinal microbial disturbance and permeability (leaky gut), and potentially obesity [33,34]. Nevertheless, a debate is still ongoing about the relevance of the disturbance of gut microbiota for metabolic disorders and whether it is a cause or an effect [35,36]. Understanding the network of pro-inflammatory and metabolite signaling, that often triggers the early stages of metabolic disorders, emphasizes the importance of the gut microbiota especially in cases of obesity following a high-fat diet [37]. Although extensive research has been performed on the interplay between the pathogenesis of obesity and the diversity of gut microbiota, a big gap is still yet to be filled. The significance and extent of gut bacterial disturbance that is enough to develop into metabolic syndrome has not yet been well explained. Moreover, there has been a long debate about the specific gut bacterial taxa that are mostly affected during conditions of obesity with sometimes contradicting opinions about some genera.

Another interesting interplay to consider in studying obesity is the gut–brain axis. Looking into the intriguing potential link between metabolic syndrome and brain health is an emerging concern that has caught the attention of nutritional microbiologists and neurologists in the past few years yet only a handful of studies have been published linking a high-fat diet to cognitive skills [38,39]. The proposed effect of obesity on cognition is believed to be a multifactorial process. Gut dysbiosis and subsequent changes in intestinal permeability and tight junction are direct manifestations of obesity that may have direct effects on brain health through increased peripheral and central inflammation caused by an excessive expression of pro-inflammatory cytokines (TNF α and IL-6) [40]. Moreover, accumulating white adipose tissue (WAT) in high-fat-induced obesity is associated with excessive production of free fatty acids in the context of immune system activation and excess expression of adipokines, including leptin [41]. The condition of low-grade inflammation is usually not limited to the periphery but may also lead to neuroinflammation. Obese subjects can experience a neuroinflammatory effect on vital parts of the brain that are in charge of learning, memory, and emotions, which is often marked by a higher expression of TNF α and IL-6 [42]. This is further worsened by the fact that the altered permeability is not usually limited to the gut but may also compromise the permeability of the blood–brain barrier (BBB) and the transport of peptides [43]. Changes in the activity of cholinergic enzymes like acetylcholinesterase (AChE) have been reported as a good indicator of brain cholinergic regulation and a direct reflection of acetylcholine concentration. Declining levels of acetylcholine are frequently associated with cognitive impairment and have been used as a biomarker for some neurodegenerative disorders [44]. Altered AChE has been indirectly linked to neuroinflammation and oxidative stress in different brain structures [40]. A close correlation between obesity and brain health can be suggested based on the microbiota–colon–brain axis in a process involving dysbiosis and neuroinflammation [40,45]. To the best of our knowledge, this study is among only a few emerging studies that link high-fat diet-induced obesity to the gut–brain axis.

The present work aimed to study the implications of obesity on gut microbiota diversity and cognitive function and its impact on plasma and brain metabolic parameters in rats. Obesity was induced in rats by feeding on high-fat (HF) and high-fat/high-sucrose (HFHS) diets. The study also used high-throughput sequencing to give a comprehensive image about the intestinal ecology in obese rats consuming a high-fat diet with or without high sugar.

## 2. Materials and Methods

### 2.1. Animals and Diets

Male Sprague–Dawley rats of average weight 117 g ± 6.94, 6 weeks of age were obtained from animal house of National Research Centre, Cairo, Egypt. Animals were kept individually in stainless steel cages; water and food were given ad libitum. The high-fat diet consisted of 56.7% fat-derived calories (beef tallow) and 34.8% carbohydrate-derived calories. The high-fat/high-sucrose diet had the same composition in addition to 30% (*w*/*v*) sucrose water [38,39]. The diets were prepared as in Table 1.

### 2.2. Design of Animal Experiment

After acclimatization for one week, eighteen rats were randomly allocated into three groups (n = 6). Group 1 served as normal control (NC) and fed on balanced diet. Group 2 high-fat (HF) and group 3 high-fat/high-sucrose (HFHS) fed on high-fat diet and high-fat/high-sucrose diet for eight weeks for induction of obesity according to Mohamed et al. [47] and Ramadan et al. [48]. Food intake and body weight were recorded weekly. For HFHS group, 30% (*w*/*v*) sucrose water was supplemented. Food and water were replenished every day. For the HFHS group, energy intake was calculated as previously mentioned by Mohamed et al. [47]. Body mass index (BMI) as obesity index for all rats was determined according to the formula BMI = body weight (g)/square of length (nose to anus cm).

**Figure 1:**
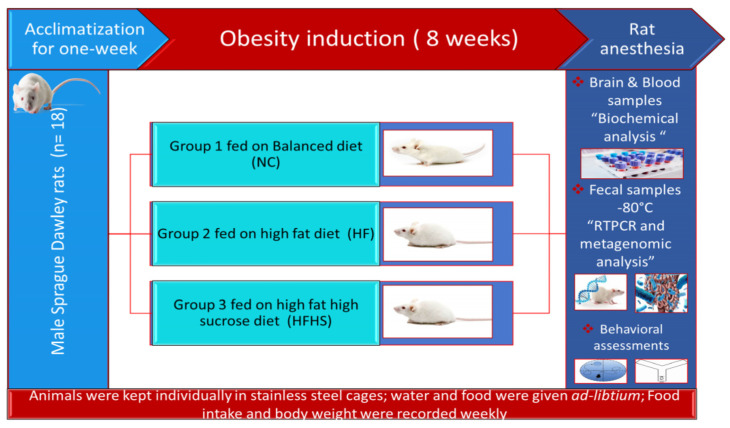
Flow chart of the animal experiment.

At the end of week 8, the impact of obesity on cognitive functions was assessed in rats using Y-maze and Morris water maze (MWM) tests. By the end of the behavioral assessment tests, animals were fasted while sucrose water of the HFHS group was replaced with tap water 12 h prior to sampling. Blood samples were collected from all rats. Fecal samples were collected from all the animals and immediately stored at −80 °C until analyzed (Figure 1). After animal anesthesia and scarification, brain was dissected for AChE analysis. All animal procedures were performed in accordance with the Ethics Committee of the National Research Centre, Cairo, Egypt, with approval number 13050203 and following the recommendations of the National Institutes of Health Guide for Care and Use of Laboratory Animals (Publication Nos. 85-23, revised 1985).

### 2.3. Determination of Acetyl Cholinesterase, Inflammatory Markers, and Metabolic and Oxidative Stress Parameters

Acetyl cholinesterase (AChE) in brain tissue and in plasma was determined (Cat No. SL002Ra, ELISA kit, SUNLOG, Hangzhou City, China). Inflammatory markers (C-reactive protein (CRP)) (ELISA kit, Catalog no. SL0202Ra, Sunlong^®^, Hangzhou City, China) and tumor necrosis factor-α (TNF-α) (ELISA kit, catalog no. SL0722Ra, Sunlong^®^, Hangzhou City, China), along with plasma leptin (ELISA kit, catalog no. SL0441Ra, Sunlong^®^, Hangzhou City, China), were estimated according to the manufacturer’s instructions.

Plasma malondialdehyde (MDA) [49] as an indicator of lipid peroxidation was determined. Catalase activity [50] was evaluated as an indicator of antioxidant status. Plasma oxidized-LDL (ox-LDL) was determined in all samples using ELISA kit (catalog no. SL0554Ra, Sunlong^®^, Hangzhou City, China). Plasma glucose [51] and plasma insulin (ELISA kit, catalog no. SL0373Ra Sunlong^®^, Hangzhou City, China) were determined. Insulin resistance was calculated based on homeostasis model assessment of insulin resistance (HOMA-IR), according to Cacho et al. [52] and using the equation:

[FPG (mmol/l) × FPI (μU/mL)]/22.5

### 2.4. Lipid Profile and Liver and Kidney Functions

Plasma levels of total cholesterol (T-ch), triglycerides (TG), high-density lipoprotein cholesterol (HDL-Ch), and low-density lipoprotein cholesterol (LDL-Ch) were all assessed using colorimetric kits. The ratio of T-Ch to HDL-Ch was calculated. Plasma levels of creatinine [53] and urea [54] were determined as indicators of kidney function, while the activity of transaminases, aspartate transaminase (AST) and alanine transaminase, (ALT) was determined for liver function [55].

### 2.5. DNA Extraction and Phylum Quantification Using Real-Time PCR 

Genomic DNA was isolated from rat feces (200 mg) using QIAamp^®^-DNA-Stool-Mini-Kit (Qiagen, Valencia, CA, USA) in accordance with instructions of manufacturer. The extracted DNA quality and concentrations were measured using NanoDrop-spectrophotometer (ThermoFisher Scientifics, Wilmington, DE, USA).

DNA samples were then analyzed to quantitatively determine Bacteroidetes and Firmicutes, representing the 2 largest phyla in the colonic microbiota, using real-time PCR. Specific oligonucleotide primers were chosen from the previous literature [56]. Specificity of the primer sequences (Table 2) was confirmed using NCBI BLAST database. Real-time PCR was performed using a Rotor-Gene^®^ MDx machine (Qiagen, Valencia, CA, USA). The qPCR reaction was performed in a total volume of 25 µL containing 2 µL gDNA, 4 µL EvaGreen^®^ master mix plus, (Solis BioDyne, Tartu, Estonia) and 0.3 µM of forward and reverse primers. The PCR reaction conditions for DNA amplification were 50 °C for 2 min, 95 °C for 15 min, 50 cycles of 15 s at 95 °C, 60 s at 60 °C, 15 s at 72 °C, and melting curve program (55–95 °C). The final counts were calculated as log10 copies from standard curves and normalized to grams of wet feces.

The DNA of *Lactobacillus plantarum* was used as the standard, and the standard curve was generated by plotted *L. plantarum* copy numbers against Ct. Primer sequences of *L. plantarum* were adapted from Haarman and Knol [57]; sequences were as follows: *L. plantarum*-F (5′tggatcacctcctttctaaggaatg3′) and *L. plantarum*-R(5′tgttctcggtttcattatgaaaaaatag3′).

### 2.6. 16S Library Preparation and Metagenomic Sequencing

The procedure took place at Macrogen (Seoul, Republic of Korea). NexteraXT library preparation-kit (Illumina, San Diego, CA, USA) with Herculase II fusion DNA polymerase (Agilent Technologies, Santa Clara, CA, USA) was used to amplify the 16S rRNA variable regions (V3 and V4). The primers used for amplification were 16S amplicon PCR forward primer 5′tcgtcggcagcgtcagatgtgtataagagacagcctacgggnggcwgcag-3′ and reverse primer 5′gtctcgtgggctcggagatgtgtataagagacaggactachvgggtatctaatcc-3′. Libraries were prepared according to 16S Metagenomic Sequencing Library Preparation Protocol (Illumina, San Diego, CA, USA, Part # 15044223, Rev. B). Indices and sequencing adapters were attached using Nextera XT index V2 kit (Illumina, San Diego, CA, USA). The final library was sequenced using the MiSeq sequencing platform (Illumina, San Diego, CA, USA) to generate 300 bp paired-end reads (600-cycle Miseq Reagent Kit v3). Raw image files obtained in the platform were processed to generate base calling through RTA (Real-Time Analysis v1.18, Illumina, San Diego, CA, USA), which was converted into FASTQ utilizing MSR (Miseq Reporter, Illumina, San Diego, CA, USA).

### 2.7. Sequence Processing and Analysis

The resulting paired-end reads were merged using Usearch -fastq_mergepairs. The raw reads were purified and trimmed using Trimmomatic version 0.38 to remove the low quality (Phred score ≤ 25), short reads, and adapters. Generation of Operational Taxonomic Units (OTUs) and mapping of reads to OTUs was then performed. All quality reads were analyzed using the Greenfield Hybrid Analysis Pipeline (GHAP) (Commonwealth Scientific and Industrial Research Organization (CSIRO), Australia, version 1.0 CSIRO. Software Collection 2017, https://doi.org/10.4225/08/59f98560eba25) was based on the Usearch tools and the Ribosomal Database Project (RDP) classifier. The Green Genes reference database was used to assign taxonomy to the OTUs. Following this, alpha diversity (Chao1, Shannon, and Simpson indices) was calculated and microbiome analysis was performed using the quantitative insights into microbial ecology version 2 (QIIME2) pipeline [58]. A sequence depth of 20,000 reads per sample was selected to analyze the alpha diversity metrics with 1000 iterations.

### 2.8. Behavioral Assessment of the Impact of Obesity on Cognitive Functions 

Y-maze test [59] and MWM test [60] were used to evaluate the impact of obesity on short-term memory, learning capacity, and visuo-spatial memory in all obese rat groups compared to the normal rat group. All behavioral assessment tests were carried out according to the methods previously described [61,62].

### 2.9. Statistical Analysis

The results of animal experiments and biochemical parameters are expressed as mean ± SE. Statistical analysis was performed using Prism software version (10). Means were compared using one-way analysis of variance ANOVA followed by Duncan’s test. Non-parametric tests (Kruskal–Wallis test followed by Mann–Whitney test) were used whenever the data were not normally distributed. Escape latency time for four days using MWM was analyzed using two-way ANOVA followed by Bonferroni test. Mean differences were considered significant at *p* ≤ 0.05.

## 3. Results

### 3.1. Impact of High-Fat/High-Sucrose Diets on Nutritional Parameters of Obese Rats

**Figure 2:**
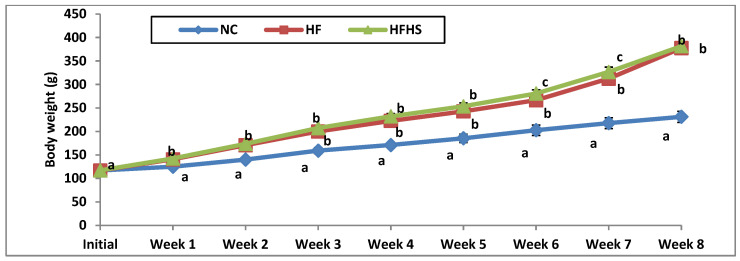
Growth curves of different rat groups during the study. NC: Normal control, HF: High-fat, HFHS: High-fat/high-sucrose. Similar letters mean non-significant difference within groups at *p* < 0.05.

The impact of a high-fat diet with or without sucrose on body weight, BMI, and food intake is summarized in Table 3. Figure 2 illustrates the growth curves of different groups during the study. The final body weight and body weight gain significantly (*p* < 0.001) increased in the HF and HFHS groups compared to the normal control group. All rats showed an increase in body weight during the study (Figure 2). Rats fed on the HF diet or HFHS diet showed a significant increase in body weight compared with the normal group from week two to the end of the study in week eight (Figure 2). The HFHS diet showed a significant increment in rat body weight compared with rats fed on the HF diet in the 6th and 7th weeks of the study (Figure 2). The BMI as an indicator of obesity significantly increased among the HF (0.683) and HFHS (0.688) groups compared to the control group (*p* < 0.001). Both diets were effective in the induction of obesity as noticed from the BMI, which was elevated more than 0.68 g/cm^2^. The food efficiency ratio showed a significant elevation in the HF and HFS groups compared to the control group (*p* < 0.001).

### 3.2. Impact of Obesity on Acetyl Cholinesterase, Oxidative Stress, Inflammation, and Hyperglycemia

**Figure 3:**
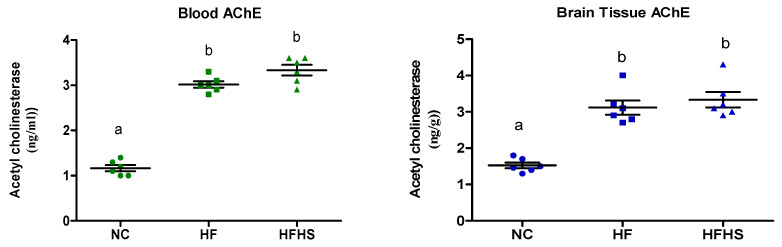
Acetyl cholinesterase in brain tissue of normal rat and obese rat groups. Similar letters mean non-significant difference within groups at *p* < 0.05.

Obese rats fed on HF and HFHS recorded a significant increase in acetyl cholinesterase in both brain tissue and plasma compared to normal rats (Figure 3). Obese rats fed on the HF and HFHS diets were observed to have significantly elevated insulin, glucose, and IR levels compared to the normal control (*p* < 0.001) as shown in Table 4. The activity of catalase significantly decreased (*p* < 0.001), while that of MDA significantly increased (*p* < 0.001) in the HF and HFHS groups, which indicated the presence of oxidative stress in obese rats. The levels of TNF-α, CRP, and leptin were significantly (*p* < 0.001) elevated in the HF and HFHS groups, as compared to the control group. The elevation of inflammatory markers is an indicator of chronic low-grade inflammation in obese rats (Table 4).

### 3.3. Impact of Obesity on Lipid Profile and Liver and Kidney Functions

Concentrations of T-Ch, TG, LDL-Ch, oxi-LDL, and TCh/HDL were significantly higher in the obese rat groups (HF and HFHS) than in the control group. HDL-Ch levels significantly decreased in the HF and HFHS groups than in the control group. Rats fed on the HF and HFHS diets showed a significant increase in the activity of AST, ALT, creatinine, and urea as compared to the control group (Table 4).

### 3.4. Impact of Obesity on Cognitive Functions

The impact of obesity on cognitive functions was evaluated through the different behavioral tests of all obese rats compared to normal rats. Obesity led to a decline in cognitive function as elucidated in the inspected Y-maze test and water maze test.

#### 3.4.1. Y–Maze Test

**Figure 4:**
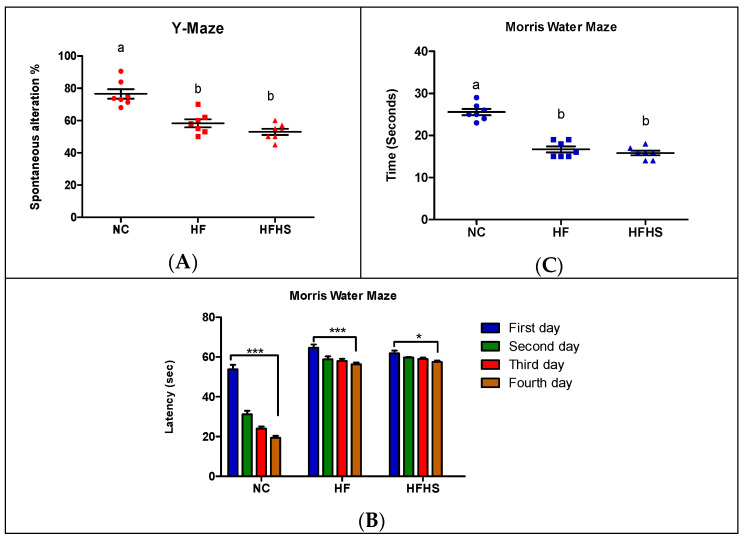
Y-maze and Morris water maze tests of normal rat and obese rat groups. (**A**,**C**) Similar letters mean non-significant difference within groups at *p* < 0.05. (**B**) * *p* > 0.05 and *** *p* > 0.001.

The Y-maze test is an indicator for short-term memory. Obesity led to a significant decrease in the percentage change seen in the obese rats by 23.8% and 30.7% in the rats fed high-fat and high-fat/high-sucrose diets, respectively (Figure 4A), compared to normal rats.

#### 3.4.2. The MWM Test

Spatial signal learning and memory of rats were assessed using the MWM test. The test was performed twice, and the average of duplicate readings for each rat group was recorded for each day of the test. On the first day, rats in all groups took about 60 s for the test to attain the platform. From the second day to the fifth day, obese rats showed a considerable reduction in the time recorded in comparison to normal rats. The results obtained from the escape latency (second) during spatial learning in the MWM test show no statistically significant difference between the experimental groups on the first day. Upon comparison between days in the experimental groups, the mean escape latency to the platform on days 2, 3, and 4 was statistically shorter than on day 1 for all groups (Figure 4B). The reduction in escape latency to the platform for obese rats was significantly lower than for normal rats. These results reveal that obesity led to a reduction in learning ability in obese rats compared to normal rats. On the fifth day, the average time spent in the target quadrant for rats fed the high-fat or high-fat/high-sucrose diets reduced by 8.9 and 9.7 s, respectively, in comparison to that recorded in the group of the normal control (Figure 4C). Both behavioral tests indicated that rats fed the high-fat/high-sucrose diet showed more of a decline in cognitive function compared with rats fed the high-fat diet only. The difference between the two groups, however, was not significant.

### 3.5. Quantification of Two Major Fecal Phyla Using Real-Time PCR

**Figure 5:**
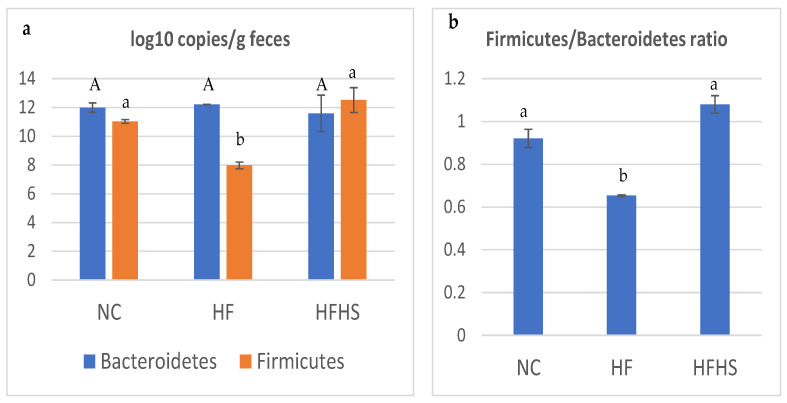
Counts of Bacteroidetes and Firmicutes phyla in rat feces quantified by qPCR and expressed as log10 copies/g wet feces and Firmicutes/Bacteroidetes ratio. (**a**) Bacteroidetes and Firmicutes count; (**b**) Firmicutes/Bacteroidetes ratio. For each phylum, bars with different letters are significantly different at *p* ≤ 0.05.

Figure 3 shows the counts of two major phyla of the rat gut microbiota among the test groups and Firmicutes/Bacteroidetes ratio. The Bacteroidetes phylum did not show significant differences between the different groups. A significant decline in the count of the Firmicutes phylum was seen in the HF group compared to the other two groups (Figure 5a). The Firmicutes/Bacteroidetes ratio decreased in the HF group, while it increased in the HFHS group compared to the normal control (Figure 5b).

### 3.6. Metagenomic Analysis

**Figure 6:**
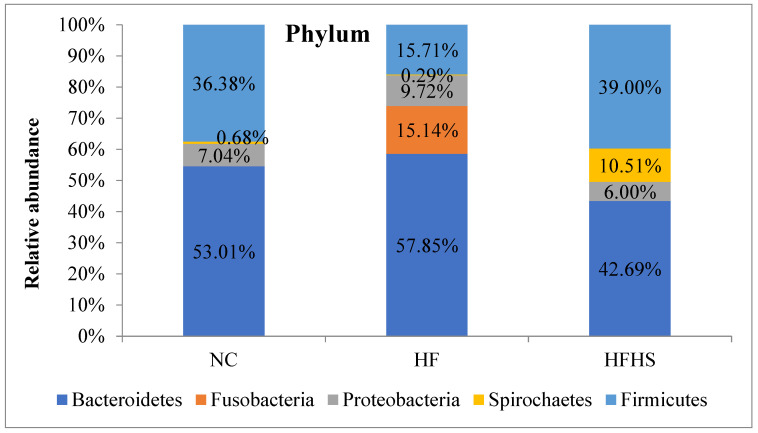
Relative abundance of microbiota at the phylum level in the guts of rats among the test groups using metagenomic analysis. NC: Normal control, HF: High-fat, HFHS: High-fat/high-sucrose.

Bacteroidetes, Firmicutes, Proteobacteria, Fusobacteria, and Spirochaetes phyla were detected (Figure 6). Proteobacteria increased in the HF group compared to the control. Fusobacteria were only detected in the HF group. The Spirochaetes and Firmicutes phyla were lower in the HF group compared to the control. A decrease in the Bacteroidetes phylum and an increase in Firmicutes and Spirochaetes were seen in the HFHS group compared to the control group. The ratio between Firmicutes and Bacteroidetes (F/B ratio) showed a decrease in the HF group and a sharp increase in the HFHS group compared to the control.

**Figure 7:**
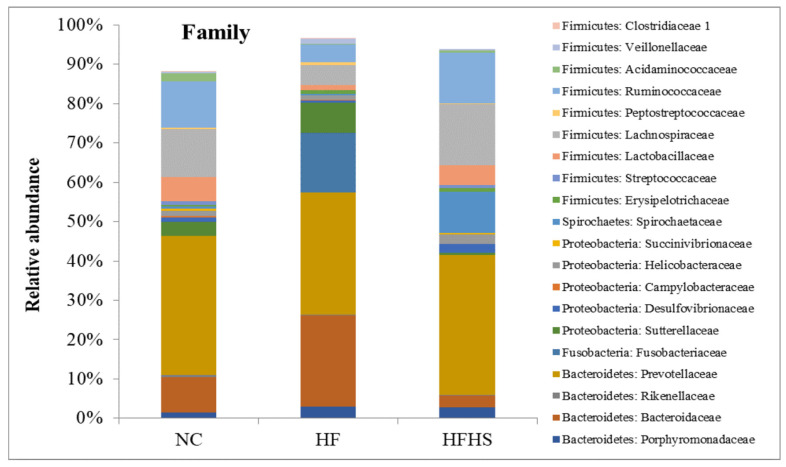
Relative abundance of microbiota at the family level in the guts of rats among the test groups using metagenomic analysis. NC: Normal control, HF: High-fat, HFHS: High-fat/high-sucrose.

At the family level (Figure 7), *Bacteroidaceae* bloomed in the HF group compared to the control. A similar abundance pattern was even seen in *Peptostreptococcaceae*. However, the abundance of *Prevotellaceae*, *Lachnospiraceae*, *Ruminococcaceae*, *and Lactobacillaceae* shrank compared to the control group. Adding sucrose to the high-fat diet in the HFHS group reversed the increase in *Bacteroidaceae* and *Peptostreptococcaceae,* but it also interestingly reversed the decrease in *Prevotellaceae*, *Lachnospiraceae*, and *Ruminococcaceae* compared to the HF group.

**Figure 8:**
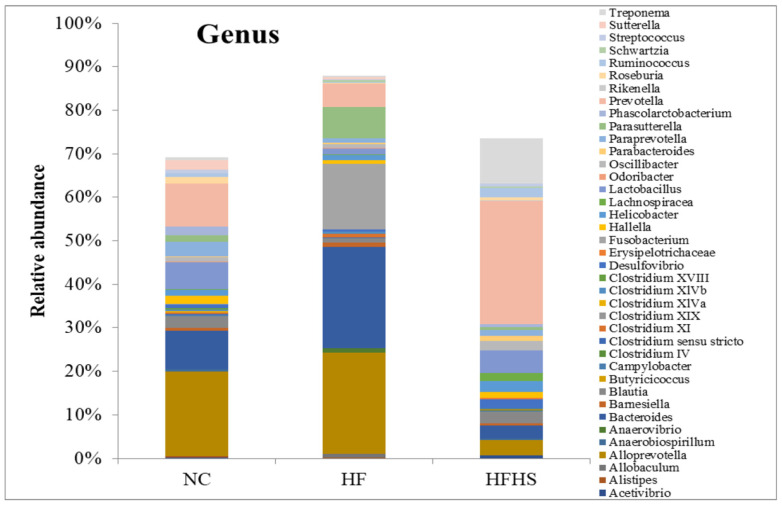
Relative abundance of microbiota at the genus level in the guts of rats among the test groups using metagenomic analysis. NC: Normal control, HF: High-fat, HFHS: High-fat/high-sucrose.

Similar relative abundance patterns were also found at the genus level with clear differences between the HF and HFHS groups. Short-chain acid (SCFA)-producing genera like *Ruminococcus*, *Lactobacillus*, *Butyricicoccus*, and *Acetivibrio* dropped in the HF group compared to the control. The same genera did not show the same decrease in the HFHS group. On the other hand, enrichment was noted in *Bacteroides* in the HF group compared to the control, while it dropped in the HFHS group. The obesity associated the *Prevotella* genus and the endotoxin-producing *Desulfovibrio* genus was highly enriched in the HFHS group, while *Alloprevotella* showed a sharp decrease in relative abundance compared to the control. The abundance of *Paraprevotella* and some *Clostridium* genera decreased equally in both the HF and HFHS groups compared to the control as shown in Figure 8.

**Figure 9:**
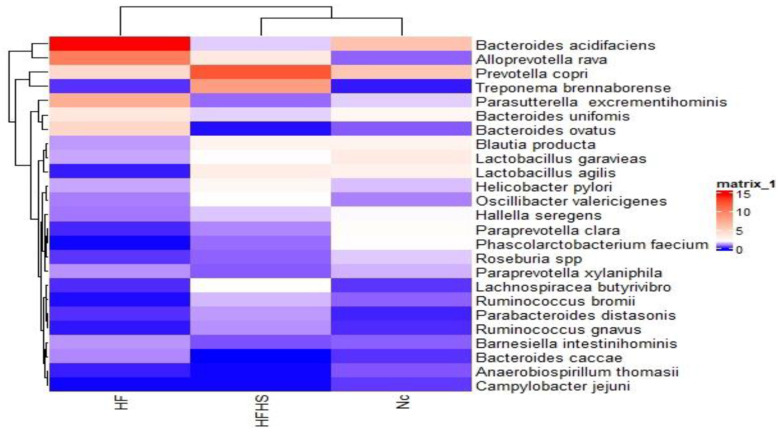
Heat map of the top 25 species differentially enriched across the test groups. NC: Normal control, HF: High-fat, HFHS: High-fat/high-sucrose.

Figure 9 shows the top 25 species that were differentially enriched among the test groups. Several species showed different enrichment levels in the HF and HFHS groups. *Lactobacillus agilis* and *Ruminococcus bromii* dropped in the HF group only, with no changes in the HFHS group compared to the control. Some SCFA-producing species that have been linked to reduced inflammation showed a sharp decrease in the HFHS group including *Bacteroides acidifaciens* and *Bacteroides ovatus*, while *Prevotella copri*, which is linked to carbohydrate metabolism, was highly enriched. *Alloprevotella rava* was enriched in both groups compared to the control. *Alloprevotella tannerae* decreased in the HF group and was not detected in the HFHS group. *Campylobacter jejuni* and *Roseburia* spp. both seemed to be highly suppressed in the HF and HFHS groups. 

## 4. Discussion

Diet-induced obesity is a global phenomenon that affects the population worldwide with manifestations at both the phenotypic and genotypic levels. While studies suggest possible forms of genetic predisposition for obesity, diet-induced obesity seems to be a growing concern due to its rapidly growing prevalence with increasing burden on the health and economic sectors [63]. A diet high in fat and/or sugar content may lead to hyperlipidemia in a process involving gut microbiota perturbation [64]. The disturbance of the intestinal bacterial balance resulting from consuming an unbalanced diet is a major risk factor in developing metabolic syndrome, which is the first step in a cascade of events that may lead to common chronic disorders including obesity [65]. In the present rat model, the induction of obesity was confirmed with significant increases in the BMI and body weight gain in both the HF and HFHS groups. Both parameters have been reported to increase in obese subjects in different animal models [66,67]. According to previous reports, long-term dietary fat intake has a direct effect on lipid accumulation and blood cholesterol [68,69]. This was reflected in our study by a significantly altered lipid profile in the HF and HFHS groups. The consumption of high sucrose in addition to the high-fat diet showed a further deterioration in the lipid profile. This was seen in the plasma T-Ch, LDL, oxi-LDL, and T-Ch/HDL-Ch concentrations being significantly higher in the HFHS group than in the HF group. The effect of sugar intake on blood cholesterol and circulating lipids has been reported in similar rat model studies as well as studies on obese patients [70,71].

Insulin resistance (IR) is a common key factor in the onset and development of metabolic syndrome and obesity [72]. Elevated blood glucose and insulin have been reported in obese patients with or without diabetes [73,74]. Similarly, in this study, the significant increase in IR and blood glucose levels was independent of sugar intake. 

C-reactive protein (CRP) is a pro-inflammatory biomarker that negatively affects leptin bioavailability and interferes with glucose metabolism by reducing insulin sensitivity [75]. This mechanism involves CRP docking to the extracellular domain of the leptin receptor [76]. In epidemiological studies, high concentrations of blood CRP and leptin were associated with high insulin resistance [77]. Obese subjects usually show high levels of plasma leptin, which reflects how leptin fails to do its job despite its high abundance [78]. This was also found in this study, with plasma CRP and leptin being significantly higher in the HF group compared to the control. Interestingly, feeding the rats a high-fat diet supplemented with high sucrose significantly elevated plasma leptin compared to the rats fed on a high-fat diet only. Since CRP production is influenced by leptin [79], these findings emphasize the impact of CRP on glucose metabolism and energy transport. TNF-α is another pro-inflammatory cytokine that has long been linked to dyslipidemia and IR [80]. The significant increase in TNF-α in both the HF and HFHS groups confirms the progression of low-grade inflammation, which is a common feature in obesity. Elevated adipokines and cytokines have been previously reported following the long-term consumption of high-fat diets with or without sugar [81,82]. This chronic condition of inflammation seems to go far and beyond, affecting other organs that are usually involved in metabolic syndrome. This can be seen in the significantly disturbed liver function enzymes in this study for both the HF and HFHS groups. We previously showed similar findings in a study on the link between steatohepatitis and a high-fat diet [26]. It has also been observed in many other dietary intervention studies [47,48,83,84].

The results of the metabolic parameters seemed to correlate well with the gut microbiota analysis. The findings from the qPCR analysis and metagenomic sequencing reflect clear abundances of microbial taxa that are usually associated with obesity, inflammation, and IR in the HF and HFHS groups. They also show remarkable suppression in some gut bacterial communities that are related to short-chain fatty acid (SCFA) production as well as bacterial families that are involved in mitigating endotoxemia, leaky gut, and inflammation. This can also be seen in the greatly reduced overall diversity of microbiota in the HF group as shown by all three alpha diversity indices. This obesity-associated feature has been repeatedly mentioned in previous studies [37,85]. Added sugar in the HFHS group, however, did not seem to similarly affect the diversity which might be due to different metabolic pathway patterns and SCFA production between the HF and HFHS groups. The counts of the Bacteroidetes and Firmicutes phyla showed similar patterns using qPCR or 16S rRNA sequencing. Metagenomics analysis, however, shed more light on the topic and provided more evidence to corroborate the biochemical and qPCR results. The higher abundance of Firmicutes combined with the low abundance of Bacteroidetes shown in the HFHS group is a common marker of metabolic syndrome and has been frequently reported in obese subjects [86,87]. The sharp increase in the F/B ratio in the same group suggests a link between high sugar intake and disturbed Firmicutes to Bacteroidetes rates. This has been previously reported in studies involving diabetic subjects and in metagenomic studies following high sugar consumption [88,89]. The lack of similar effects in the HF group can be attributed to the fact that the Firmicutes phylum contains different genera of beneficial gut bacteria as well as adaptable pathogens. Moreover, a reduced F/B ratio in the HF group brings to light a common debate about if this ratio is a true and reliable marker for obesity [90]. The appearance of Fusobacteria in the HF group confirms the previous reports about this obesity-associated phylum [91].

Although both the HF and HFHS groups showed clear signs of altered bacterial communities compared to the control, this alteration has not been consistent between the two groups and appeared to be very selective for the HFHS group. This is clearly observed in the enrichment patterns of some beneficial gut genera. *Lactobacillus*, *Butyricicoccus*, *Rhuminococcus*, and some *Clostridium* genera are known SCFA producers that are associated with gut health and a state of eubiosis (which is the flourishing of beneficial bacteria in the gut) [92,93]. The big reduction in these genera in the HF group confirms the idea about the gut dysbiosis associated with IR and obesity [94]. The gut of obese patients has been reported to harbor high levels of succinate-producing gut genera like *Prevotella* and other genera that are not confirmed producers but have been repeatedly correlated with gut succinate production like *Peptostreptococcus* [95], which justifies the reported elevated levels of fecal succinic acid that has been linked to obesity and chronic inflammation [96]. Our study shows a higher relative abundance of *Prevotella* in the HFHS group compared to the control which adds to the evidence connecting this genus to obesity and IR [97]. A concurrent big suppression in the SCFA-producing *Alloprevotella* in the HFHS group also supports the claims of an inverse relationship between some obesity-associated genera and some SCFA producers in gut dysbiosis [98]. This idea is even further supported by the high enrichment of the endotoxin-producing *Desulfovibrio* in the HFHS group. *Bacteroides* is a genus that contains both beneficial and obesity-associated subtypes (Bact2 enterotype) [99,100]. This fact may explain the inconsistency in its relative abundance between the HF and HFHS groups.

Some gut bacterial species that have been previously linked to inflammation, oxidative stress, and metabolic syndrome showed a clear enrichment in the HF and HFHS groups, while other beneficial species that have been correlated with improving inflammation and endotoxemia were greatly suppressed. *Bacteroides acidifaciens* and *Bacteroides ovatus* were drastically compromised by the high-fat/high-sucrose diet. The two species are associated with IR and endotoxemia-alleviated conditions [101]. The same can be said about *Roseburia* spp., which is a marker of eubiosis [102]. *Alloprevotella rava*’s remarkable increase in both the HF and HFHS groups may be attributed to its association with dyslipidemia although this claim is highly arguable according to some studies [103,104]. A remarkable increase in the *Prevotella copri* species in the high-fat/high-sucrose diet group only opens a debate about its relevance to carbohydrate and lipid metabolism as well as gut inflammation [105]. It is also noteworthy to mention the decrease in *Lactobacillus agilis* and *Rhuminococcus bromii* in the HF group but not in the HFHS group as further evidence of possible differential metabolic pathways between the two groups. Recent studies for some of our research team members emphasized the correlation between the progression of metabolic disorders like dyslipidemia, diabesity, and fatty liver and the development of dysbiosis and metabolic endotoxemia resulting in disruption of gut tight junction proteins and leading to increased gut permeability (leaky gut) [26].

These findings further emphasize the main mechanisms associating obesity with changes in the gut microbiome. Perturbations in essential bacterial taxa and SCFA production and metabolism appear to be key mechanisms in the process due to a high fat intake. A leaky gut caused by a loss of tight junction proteins in the intestinal barrier usually follows leading to the activation of various cytokines through low-grade inflammation in the gut as well as systemically and peripherally. Abdominal fat accumulation is also another mechanism that seems to contribute to chronic inflammation and IR through disturbed fatty acid metabolism both locally and systemically. This suggests that dysbiosis might be an effect of obesity rather than a cause. This cycle of cause and effect, however, needs further investigation.

The findings of brain performance and memory tests in this study further corroborated the claims of dysbiosis and the impact of obesity on brain health. Cognitive impairment following a long-term consumption of a high-fat diet has not yet been well studied. This is in part due to the complex nature of brain functions and the diverse ways in which cognitive decline can manifest itself. Common learning and memory skills have been tested in obese subjects with different outcomes. Some recent studies suggested a possible correlation between high fat consumption and neuroinflammation in the amygdala and the hippocampus, the main brain structures regulating behavior, learning, and memory [42]. In another study on spatial memory in rats, dysbiosis following a high-fat diet did not seem to affect cognition [38]. The hypothesized effect of a high-fat diet on cognition can be explained through dysbiosis. Obesity-induced dysbiosis in the gut is usually marked by a reduced expression of the gut junction proteins ZO-1 and occludin which in turn leads to increased serum lipopolysaccharide (LPS) through a ‘leaky gut’ membrane [106], and the resulting low-grade systemic inflammation and activated pro-inflammatory cytokines have the potential of affecting the BBB and triggering neuroinflammation in vital brain parts, particularly the hippocampus and the amygdala [107,108]. This effect was reflected in this study by a significant drop in the short-term and visuo-spatial memory in both the HF and HFHS groups.

Cholinergic activity is a good measure of brain health and has been used as a diagnostic tool for many neurodegenerative diseases. AChE is a regulatory enzyme that can be used as a good measure of acetylcholine activity which is closely related to learning and memory in brain structures through regulating synaptic impulse transmission [109]. The elevated levels of AChE observed in this study in both the plasma and brain tissue can be explained in light of previous studies that demonstrated the effect of a high-fat diet on brain health through the down-regulation of acetylcholine [44]. Although the behavior of AChE in obesity has not always been consistent according to some studies and it seems to be dependent on the brain structure under study [110], there is a clear impact of diet-induced obesity on the brain functions regulated by the cholinergic system. 

The relationship between gut microbiota and cognition is well established, and the gut–brain axis has been subjected to extensive research. However, the delicate link between obesity due to high fat and cognition is still unclear but dysbiosis seems to be the main driving factor. The direct effect of dysbiosis is a loss of diversity in the gut microbiota which reflects in lower cognitive functions. An increased F/B ratio has been previously associated with reduced learning skills [111]. The bacterial taxa affected by the altered eubiosis in the gut can involve SCFA-producing bacteria. This group of bacteria is responsible for the production of tight junction proteins and thus improves gut permeability and may prevent brain impairment and peripheral inflammation from leaking cytokines. Disturbance in the balance of these bacterial taxa can mediate the progress of systemic and neuroinflammation [112]. Similar findings are seen in this study where the same group of gut bacteria have been compromised by the HF diet. More evidence comes from the fact that the same gut bacterial taxa associated with inflammation and IR have also been linked to memory and cognitive impairment [113,114]. Our results show more consistency for both the HF and HSHF diets in the brain health tests than in the gut metagenomic analysis, which suggests that other mechanisms might be involved along with dysbiosis. The selective dysbiosis in the high-sugar diet group with consistent cognitive impairment makes it difficult to pinpoint particular bacterial taxa as the sole triggers of cognitive impairment. It rather suggests a more multifactorial mechanism involving low-grade inflammation along with adipokine expression and BBB-altered permeability. Further research is needed in this regard to define particular mechanisms and bacterial taxa.

A few lessons learned from this study can be helpful for deriving practical applications for patients at different severity degrees of obesity. Management strategies should focus on counteracting the decline in cognitive function and not only on the metabolic consequences of the disease. Lifestyle changes, especially regular physical activity, can be preventive measures for healthy individuals as well as successful approaches to reinforce cognitive function in metabolically unhealthy patients regardless of their age group [115,116,117,118].

## 5. Conclusions

High-fat and high-fat/high-sucrose diets were effective as obesity inducers as shown by the elevation of the BMI. Also, both groups showed an increase in acetyl cholinesterase in the brain tissue and plasma in association with dyslipidemia and an elevation of oxidative stress, insulin resistance, and inflammatory markers and an alteration in liver and kidney functions. Obesity led to cognitive impairment in the form of declined learning skills, short-term memory, and visuo-spatial memory. According to the metagenomic analysis, gut bacterial communities that are involved in mitigating endotoxemia, leaky gut, and inflammation showed remarkable suppression in the HF and HFHS groups. Some gut bacterial species that are linked to inflammation, oxidative stress, and metabolic syndrome showed a clear enrichment in the HF and HFHS groups. Overall, adding sugar to the high-fat diet in the HFHS group did not seem to consistently and greatly worsen the dysbiosis found in the HF group. Rather, it even restored a few bacterial taxa to nearly normal levels. Future prospects in this area should include multi-omics integration studies through combining metagenomic, metabolomic, proteomic, and transcriptomic analysis datasets. This would provide a more comprehensive image about the interplay between gut microbiota, diet-induced obesity, and cognitive functions. This study also sets the foundation for future work using more rigorous statistical analyses as well as a functional pathway analysis and metagenomic biomarker discovery analysis.

Study limitations: One limitation of the present study was the small sample size in the test groups and the use of only male rats. The study also did not determine the short-chain fatty acids produced in obese rats compared to normal rats. Another limitation was the absence of dietary interventions to reduce obesity and its impact on cognitive function. We are currently carrying out an experiment for studying the potential preventive role of dietary supplements in the reduction in obesity and their effect on cognitive function in rats using a bigger sample size. Our next step is to move to clinical trials based on the findings of the animal experiments.

## Figures and Tables

**Table 1:** Composition of balanced, high-fat and high-fat/high-sucrose diets (g/100 g).

	Balanced Diet	High-Fat Diet	High-Fat/High-Sucrose Diet
Macro- and micronutrients			
Protein	10	10	10
Fat	10	35	35
Carbohydrate	70.5	50.5	50.5
AIN Mineral mix	3.5	3.5	3.5
AIN Vitamin mix	1	1	1
Cellulose	5	-	-
Ingredients			
* Casein	12	12	12
Corn oil	10	-	-
Beef tallow	-	35	35
Sucrose	22.83	22.83	22.83
Starch	45.67	25.67	25.67
Additional supplement	-	-	30% (*w*/*v*) sucrose water
** Total Calories (kCal/g)	4.20	** 5.57	5.57 + 1.2 Kcal/mL from sucrose water

*: A total of 12 g Casein was estimated to contain 10 g protein using AOAC [46]. **: A total of 56.7% calories from fat.

**Table 2:** Oligonucleotide primer sequences.

Primer Name	Primer Sequence 5′-3′	Annealing Temperature	Amplicon Size (bp)	Reference
BactF BactR	CATGTGGTTTAATTCGATGAT AGCTGACGACAACCATGCAG	60 °C 60 °C	126	[56]
FirmF FirmR	ATGTGGTTTAATTCGAAGCA AGCTGACGACAACCATGCAC	60 °C 60 °C	126	[56]

**Table 3:** Nutritional parameters among different experimental groups.

Parameters	NC	HF	HFHS
Initial Weight (kg)	117.00 ^a^ ± 1.71	116.83 ^a^ ± 4.61	117.17 ^a^ ± 1.78
Final Weight (kg)	231.33 ^a^ ± 11.76	378.33 ^b^ ± 4.94	381.77 ^b^ ± 7.92
Body Weight Gain (g)	114.33 ^a^ ± 11.61	261.50 ^b^ ± 7.73	264.50 ^b^ ± 8.56
BMI (g/cm^2^)	0.427 ^a^ ± 0.024	0.683 ^b^ ± 0.012	0.688 ^b^ ± 0.011
Total Food Intake (g)	900.00 ^a^ ± 5.77	945.83 ^b^ ± 5.23	955.83 ^b^ ± 6.11
Food Efficiency Ratio	0.13 ^a^ ± 0.01	0.276 ^b^ ± 0.008	0.277 ^b^ ± 0.008
Water Intake/Sucrose Water Intake (mL/day)	41.2 ^a^ ± 0.735	41.5 ^a^ ± 0.769	40.6 ^a^ ± 0.873

Values are mean ± SE, n = 6. Within the same row, means with different letters are significantly different at *p* ≤ 0.05. NC: Normal control, HF: High-fat, HFHS: High-fat/high-sucrose, BMI: Body mass index.

**Table 4:** The effect of HS and HFS diets on different biochemical parameters in plasma.

Parameters	NC	HF	HFHS
Insulin (µg/L)	6.47 ^a^ ± 0.09	12.50 ^b^ ± 0.25	13.37 ^b^ ± 0.43
Glucose (mg/dL)	70.49 ^a^ ± 1.72	109.74 ^b^ ± 2.76	114.67 ^b^ ± 3.13
IR	1.13 ^a^ ± 0.04	3.38 ^b^ ± 0.09	3.79 ^b^ ± 0.18
**Oxidative stress status**			
MDA (nmol/mL)	5.97 ^a^ ± 0.10	16.50 ^b^ ± 0.43	17.67 ^b^ ± 0.76
CAT (U/L)	609.35 ^a^ ± 10.46	312.05 ^b^ ± 6.94	290.83 ^b^ ± 4.55
**Inflammatory markers**			
TNFα (pg/mL)	13.72 ^a^ ± 0.16	29.00 ^b^ ± 0.73	30.50 ^b^ ± 0.56
CRP (ng/mL)	2.77 ^a^ ± 0.07	5.33 ^b^ ± 0.09	5.97 ^b^ ± 0.12
Leptin (ng/mL)	12.83 ^a^ ± 0.32	26.60 ^b^ ± 0.42	30.36 ^c^ ± 0.60
**Lipid profile**			
TCh (mg/dL)	72.15 ^a^ ± 2.03	144.61 ^b^ ± 3.08	168.33 ^c^ ± 3.78
TG (mg/dL)	69.40 ^a^ ± 1.39	117.77 ^b^ ± 2.97	134.17 ^b^ ± 3.07
HDL	43.00 ^a^ ± 0.52	27.17 ^b^ ± 0.60	26.50 ^b^ ± 0.76
LDL	18.83 ^a^ ± 0.48	87.50 ^b^ ± 2.14	112.67 ^c^ ± 3.84
TCh/HDL	1.68 ^a^ ± 0.04	5.35 ^b^ ± 0.22	6.38 ^c^ ± 0.24
Oxi LDL (pg/mL)	26.45 ^a^ ± 0.56	44.23 ^b^ ± 1.07	47.51 ^c^ ± 0.89
**Liver and kidney functions**			
AST (IU/L)	40.23 ^a^ ± 0.79	53.33 ^b^ ± 1.54	59.67 ^c^ ± 1.31
ALT (IU/L)	19.38 ^a^ ± 0.44	28.17 ^b^ ± 0.79	31.67 ^c^ ± 1.28
Urea (mg/dL)	24.73 ^a^ ± 0.57	34.33 ^b^ ± 0.80	36.33 ^b^± 0.67
Creatinine (mg/dL)	0.64 ^a^ ± 0.01	0.86 ^b^ ± 0.03	0.94 ^b^ ± 0.02

Values are mean ± SE, n = 6. Within the same row, means with different letters are significantly different at *p* ≤ 0.05. NC: Normal control, HF: High fat, HFHS: High-fat/high-sucrose, BMI: Body mass index.

## Data Availability

All data generated or analyzed during this study are included in this published article.

## References

[1] Ahima R.S. (2024). Overview of metabolic syndrome. Metabolic Syndrome: A Comprehensive Textbook.

[2] McCracken E., Monaghan M., Sreenivasan S. (2018). Pathophysiology of the metabolic syndrome. Clin. Dermatol..

[3] Kaur J. (2014). Assessment and screening of the risk factors in metabolic syndrome. Med. Sci..

[4] Mohamed S.M., Shalaby M.A., El-Shiekh R.A., El-Banna H.A., Emam S.R., Bakr A.F. (2023). Metabolic syndrome: Risk factors, diagnosis, pathogenesis, and management with natural approaches. Food Chem. Adv..

[5] van de Vyver M. (2023). Immunology of chronic low-grade inflammation: Relationship with metabolic function. J. Endocrinol..

[6] Chassaing B., Gewirtz A.T. (2014). Gut microbiota, low-grade inflammation, and metabolic syndrome. Toxicol. Pathol..

[7] Gardner C.D., Trepanowski J.F., Del Gobbo L.C., Hauser M.E., Rigdon J., Ioannidis J.P.A., Desai M., King A.C. (2018). Effect of low-fat vs low-carbohydrate diet on 12-month weight loss in overweight adults and the association with genotype pattern or insulin secretion: The DIETFITS randomized clinical trial. JAMA.

[8] De Lorenzo A., Gratteri S., Gualtieri P., Cammarano A., Bertucci P., Di Renzo L. (2019). Why primary obesity is a disease?. J. Transl. Med..

[9] Wharton S., Lau D.C.W., Vallis M., Sharma A.M., Biertho L., Campbell-Scherer D., Adamo K., Alberga A., Bell R., Boulé N. (2020). Obesity in adults: A clinical practice guideline. Can. Med Assoc. J..

[10] WHO Regional Office for Africa, Obesity. https://www.afro.who.int/health-topics/obesity.

[11] Keaver L., Webber L., Dee A., Shiely F., Marsh T., Balanda K., Perry I.J., Perry I. (2013). Application of the UK foresight obesity model in Ireland: The health and economic consequences of projected obesity trends in Ireland. PLoS ONE.

[12] WHO (2016). Global Report on Diabetes.

[13] Elmaleh-Sachs A., Schwartz J.L., Bramante C.T., Nicklas J.M., Gudzune K.A., Jay M. (2023). Obesity management in adults: A review. Jama.

[14] Wiciński M., Gębalski J., Gołębiewski J., Malinowski B. (2020). Probiotics for the Treatment of Overweight and Obesity in Humans—A Review of Clinical Trials. Microorganisms.

[15] Chaiyasut C., Sivamaruthi B.S., Kesika P., Khongtan S., Khampithum N., Thangaleela S., Peerajan S., Bumrungpert A., Chaiyasut K., Sirilun S. (2021). Synbiotic Supplementation Improves Obesity Index and Metabolic Biomarkers in Thai Obese Adults: A Randomized Clinical Trial. Foods.

[16] Marques-Vidal P., Velho S., Waterworth D., Waeber G., von Känel R., Vollenweider P. (2012). The association between inflammatory biomarkers and metabolically healthy obesity depends of the definition used. Eur. J. Clin. Nutr..

[17] Barazzoni R., Cappellari G., Ragni M., Nisoli E. (2018). Insulin resistance in obesity: An overview of fundamental alterations. Eat. Weight Disord.-Stud. Anorex. Bulim. Obes..

[18] Newens K.J., Walton J. (2016). A review of sugar consumption from nationally representative dietary surveys across the world. J. Hum. Nutr. Diet..

[19] Malik V.S., Hu F.B. (2022). The role of sugar-sweetened beverages in the global epidemics of obesity and chronic diseases. Nat. Rev. Endocrinol..

[20] Bray G.A. (2012). Fructose and risk of cardiometabolic disease. Curr. Atheroscler. Rep..

[21] Assy N., Nasser G., Kamayse I., Nseir W., Beniashvili Z., Djibre A., Grosovski M. (2008). Soft drink consumption linked with fatty liver in the absence of traditional risk factors. Can. J. Gastroenterol..

[22] Abid A., Taha O., Nseir W., Farah R., Grosovski M., Assy N. (2009). Soft drink consumption is associated with fatty liver disease independent of metabolic syndrome. J. Hepatol..

[23] Lin W.T., Kao Y.H., Li M.S., Luo T., Lin H.Y., Lee C.H., Seal D.W., Hu C.Y., Chen L.S., Tseng T.S. (2023). Sugar-Sweetened Beverages Intake, Abdominal Obesity, and Inflammation among US Adults without and with Prediabetes—An NHANES Study. Int. J. Environ. Res. Public Health.

[24] Lee I.S., Shin G., CHoUe R. (2010). Shifts in diet from high fat to high carbohydrate improved levels of adipokines and pro-inflammatory cytokines in mice fed a high-fat diet. Endocr. J..

[25] Jamar G., Ribeiro D.A., Pisani L.P. (2021). High-fat or high-sugar diets as trigger inflammation in the microbiota-gut-brain axis. Crit. Rev. Food Sci. Nutr..

[26] Al-Okbi S.Y., Amin M.A., Mohamed A.E., Edris A.E., Sharaf O.M., Mabrok H.B., Ramadan A.A. (2020). Basil Essential Oil and Its Nanoemulsion Mitigate Non-Alcoholic Steatohepatitis in Rat Model with Special Reference to Gut Microbiota. J. Oleo Sci..

[27] Wang P.X., Deng X.R., Zhang C.H., Yuan H.J. (2020). Gut microbiota and metabolic syndrome. Chin. Med. J..

[28] Thomas M.S., Blesso C.N., Calle M.C., Chun O.K., Puglisi M., Fernandez M.L. (2022). Dietary Influences on Gut Microbiota with a Focus on Metabolic Syndrome. Metab. Syndr. Relat. Disord..

[29] Crudele L., Gadaleta R.M., Cariello M., Moschetta A. (2023). Gut microbiota in the pathogenesis and therapeutic approaches of diabetes. EBioMedicine.

[30] Hemmati M., Kashanipoor S., Mazaheri P., Alibabaei F., Babaeizad A., Asli S., Eslami M. (2023). Importance of gut microbiota metabolites in the development of cardiovascular diseases (CVD). Life Sci..

[31] Mohamed D.A., El-Sayed H.S., Abd El-Gawad M.A.M., Abdelgayed S.S., Hamed I.M., Mohamed R.S. (2021). Characterization of stirred yoghurt enriched with probiotics and beetroot and its therapeutic potential in experimental type 2 diabetes. Acta Sci. Pol. Technol. Aliment..

[32] Al-Okbi S.Y., Mohamed D.A., Hamed T.E.S., Abd El Khalek A.B., Mohammed S.E. (2019). Role of probiotic mixture with and without green tea extract in prevention of hepatorenal syndrome in rat model. Pak. J. Biol. Sci..

[33] Cavallari J.F., Schertzer J.D. (2017). Intestinal microbiota contributes to energy balance, metabolic inflammation, and insulin resistance in obesity. J. Obes. Metab. Syndr..

[34] Amabebe E., Robert F.O., Agbalalah T., Orubu E.S. (2020). Microbial dysbiosis-induced obesity: Role of gut microbiota in homoeostasis of energy metabolism. Br. J. Nutr..

[35] Bourrat P. (2018). Have causal claims about the gut microbiome been over-hyped?. BioEssays.

[36] Walter J., Armet A.M., Finlay B.B., Shanahan F. (2020). Establishing or exaggerating causality for the gut microbiome: Lessons from human microbiota-associated rodents. Cell.

[37] Malesza I.J., Malesza M., Walkowiak J., Mussin N., Walkowiak D., Aringazina R., Mądry E. (2021). High-fat, western-style diet, systemic inflammation, and gut microbiota: A narrative review. Cells.

[38] Deshpande N.G., Saxena J., Pesaresi T.G., Carrell C.D., Ashby G.B., Liao M.K., Freeman L.R. (2019). High fat diet alters gut microbiota but not spatial working memory in early middle-aged Sprague Dawley rats. PLoS ONE.

[39] Nguyen T.D., Hallenius F.F., Lin X., Nyman M., Prykhodko O. (2020). Monobutyrin and monovalerin affect brain short-chain fatty acid profiles and tight-junction protein expression in apoe-knockout rats fed high-fat diets. Nutrients.

[40] Olsthoorn L., Vreeken D and Kiliaan A.J. (2021). Gut Microbiome, Inflammation, and Cerebrovascular Function: Link Between Obesity and Cognition. Front. Neurosci..

[41] Asghar A., Sheikh N. (2017). Role of immune cells in obesity induced low grade inflammation and insulin resistance. Cell. Immunol..

[42] Guillemot-Legris O., Muccioli G.G. (2017). Obesity-induced neuroinflammation: Beyond the hypothalamus. Trends Neurosci..

[43] Rhea E.M., Salameh T.S., Logsdon A.F., Hanson A.J., Erickson M.A., Banks W.A. (2017). Blood-Brain Barriers in Obesity. AAPS J..

[44] Martinelli I., Tayebati S.K., Roy P., Micioni Di Bonaventura M.V., Moruzzi M., Cifani C., Amenta F., Tomassoni D. (2022). Obesity-Related Brain Cholinergic System Impairment in High-Fat-Diet-Fed Rats. Nutrients.

[45] Zhang P., Yu Y., Qin Y., Zhou Y., Tang R., Wang Q., Li X., Wang H., Weston-Green K., Huang X.F. (2019). Alterations to the microbiota-colon-brain axis in high-fat-diet-induced obese mice compared to diet resistant mice. J. Nutr. Biochem..

[46] AOAC (2023). Official Methods of Analysis of the Association of Official Analytical Chemists.

[47] Mohamed D.A., Mohamed R.S., Fouda K. (2020). Anti-inflammatory potential of chia seeds oil and mucilage against adjuvant induced arthritis in obese and non-obese rats. J. Basic Clin. Physiol. Pharmacol..

[48] Ramadan N.S., El-Sayed N.H., El-Toumy S.A., Mohamed D.A., Abdel Aziz Z., Marzouk M.S., Esatbeyoglu T., Farag M.A., Shimizu K. (2022). Anti-Obesity Evaluation of *Averrhoacarambola*, L. Leaves and Assessment of Its Polyphenols as Potential α-Glucosidase Inhibitors. Molecules.

[49] Satoh K. (1978). Serum lipid peroxide in cerebrovascular disorders determined by a new colorimetric method. Clin. Chim. Acta.

[50] Aebi H. (1984). Catalase in vitro. Methods Enzymol..

[51] Trinder P. (1969). Determination of glucose in blood using glucose oxidase with an alternative oxygen acceptor. Ann. Clin. Biochem..

[52] Cacho J., Sevillano J., de Castro J., Herrera E., Ramos M.P. (2008). Validation of simple indexes to assess insulin sensitivity during pregnancy in Wistar and Sprague-Dawley rats. Am. J. Physiol. Endocrinol. Metab..

[53] Bartles H., Bohmer M., Heierli C. (1972). Serum creatinine determination without protein precipitation. Clin. Chim. Acta.

[54] Fawcett J.K., Scott J.E. (1960). A rapid and precise method for the determination of urea. J. Clin. Pathol..

[55] Reitman S., Frankel S. (1957). Colorimetric methods for aspartate and alanine aminotransferase. Am. J. Clin. Pathol..

[56] Guo X., Xia X., Tang R., Zhou J., Zhao H., Wang K. (2008). Development of a real-time PCR method for Firmicutes and Bacteroidetesin feces and its application to quantify intestinal population of obese and lean pigs. Lett. Appl. Microbiol..

[57] Haarmon M., Knol J. (2006). Quantitative real-time PCR analysis of fecal Lactobacillus species in infants receiving prebiotic infant formula. Appl. Environ. Microbiol..

[58] Caporaso J.G., Kuczynski J., Stombaugh J., Bittinger K., Bushman F.D., Costello E.K., Fierer N., Peña A.G., Goodrich J.K., Gordon J.I. (2010). QIIME allows analysis of high-throughput community sequencing data. Nat. Methods.

[59] Luszczki J.J., Wojcik-Cwikla J., Andres M.M., Czuczwar S.J. (2005). Pharmacological and behavioral characteristics of interactions between vigabatrin and conventional antiepileptic drugs in pentylenetetrazole induced seizures in mice: An isobolographic analysis. Neuropsychopharmacology.

[60] D’Hooge R., De Deyn P.P. (2001). Applications of the Morris water maze in the study of learning and memory. Brain Res. Rev..

[61] Mohamed D., El-Shamarka M., Abdelgayed S., Mohamed R. (2021). Protective effect of dietary supplements against streptozotocin induced Alzheimer’s disease in mice. J. Herbmed Pharmacol..

[62] Mohamed D., Fouda K., Mabrok H., El-Shamarka M., Hamed I. (2024). Sourdough bread as Nutritional Intervention Tool for Improvement of Cognitive Dysfunction in Diabetic Rats. BMC Nutr..

[63] Concepción-Zavaleta M.J., Quiroz-Aldave J.E., Durand-Vásquez M.D.C., Gamarra-Osorio E.R., Valencia de la Cruz J.D.C., Barrueto-Callirgos C.M., Puelles-León S.L., Alvarado-León E.d.J., Leiva-Cabrera F., Zavaleta-Gutiérrez F.E. (2024). A comprehensive review of genetic causes of obesity. World J. Pediatr..

[64] Lu C., Sun T., Li Y., Zhang D., Zhou J., Su X. (2017). Modulation of the gut microbiota by krill oil in mice fed a high-sugar high-fat diet. Front. Microbiol..

[65] Sclafani A. (1984). Animal models of obesity: Classification and characterization. Int. J. Obes..

[66] Torres-Rovira L., Astiz S., Caro A., Lopez-Bote C., Ovilo C., Pallares P., Perez-Solana M.L., Sanchez-Sanchez R., Gonzalez-Bulnes A. (2012). Diet-induced swine model with obesity/leptin resistance for the study of metabolic syndrome and type 2 diabetes. Sci. World J..

[67] Ventura L.L., Fortes N.C., Santiago H.C., Caliari M.V., Gomes M.A., Oliveira D.R. (2017). Obesity-induced diet leads to weight gain, systemic metabolic alterations, adipose tissue inflammation, hepatic steatosis, and oxidative stress in gerbils (*Meriones unguiculatus*). PeerJ.

[68] Zhu T., Zhao J., Zhuo S., Hu Z., Ouyang S., Wunier, Yu S., Chen Y., Li Y., Le Y. (2021). High Fat Diet and High Cholesterol Diet Reduce Hepatic Vitamin D-25-Hydroxylase Expression and Serum 25-Hydroxyvitamin D3 Level through Elevating Circulating Cholesterol, Glucose, and Insulin Levels. Mol. Nutr. Food Res..

[69] Magri-Tomaz L., Melbouci L., Mercier J., Ou Y., Auclair N., Lira F.S., Lavoie J.M., St-Pierre D.H. (2018). Two weeks of high-fat feeding disturb lipid and cholesterol molecular markers. Cell Biochem. Funct..

[70] de María Márquez Álvarez C., Gómez-Crisóstomo N.P., De la Cruz-Hernández E.N., Zazueta C., Aguilar-Gamas C.F., Martínez-Abundis E. (2023). Differential disruption on glucose and insulin metabolism in two rat models of diet-induced obesity, based on carbohydrates or lipids. Mol. Cell. Biochem..

[71] Stanhope K.L., Havel P.J., Goran M.I., Tappy L., Lê K. (2015). Mechanisms by which dietary sugars influence lipid metabolism, circulating lipids and lipoproteins, and cardiovascular risk. Dietary Sugars and Health.

[72] Shimi G., Sohouli M.H., Ghorbani A., Shakery A., Zand H. (2024). The interplay between obesity, immunosenescence, and insulin resistance. Immun. Ageing.

[73] Nawai F., Syauqy A., Pramono A. (2024). Correlation of lipid profile, glucose, and body composition on insulin resistance in overweight and obese subjects. AcTion Aceh Nutr. J..

[74] Cui D.Y., Zhang C., Chen Y., Qian G.Z., Zheng W.X., Zhang Z.H., Zhang Y., Zhu P. (2024). Associations between non-insulin-based insulin resistance indices and heart failure prevalence in overweight/obesity adults without diabetes mellitus: Evidence from the NHANES 2001–2018. Lipids Health Dis..

[75] Li K., Hu L., Li X., Yuan Z., He J., Liu D., Yang G., Yuan L. (2023). Effect of C-reactive protein deficiency on insulin resistance reversal in rats with polycystic ovary syndrome through augmented leptin action. Diabetol. Metab. Syndr..

[76] Sudhakar M., Silambanan S., Chandran A.S., Prabhakaran A.A., Ramakrishnan R. (2018). C-Reactive Protein (CRP) and Leptin Receptor in Obesity: Binding of Monomeric CRP to Leptin Receptor. Front. Immunol..

[77] Wang X., Bao W., Liu J., Ouyang Y.Y., Wang D., Rong S., Xiao X., Shan Z.L., Zhang Y., Yao P. (2013). Inflammatory markers and risk of type 2 diabetes: A systematic review and meta-analysis. Diabetes Care.

[78] Searpace P.L., Zhang Y. (2009). Leptin resistance: A predisposing factor for diet induced obesity. Am. J. Physiol. Integr. Comp. Physiol..

[79] Hribal M.L., Fiorentino T.V., Sesti G. (2014). Role of C reactive protein (CRP) in leptin resistance. Curr. Pharm. Des..

[80] Patel R., Palit S.P., Rathwa N., Ramachandran A.V., Begum R. (2019). Genetic variants of tumor necrosis factor-α and its levels: A correlation with dyslipidemia and type 2 diabetes susceptibility. Clin. Nutr..

[81] Church J.S., Renzelman M.L., Schwartzer J.J. (2022). Ten-week high fat and high sugar diets in mice alter gut-brain axis cytokines in a sex-dependent manner. J. Nutr. Biochem..

[82] Weiner J., Dommel S., Gebhardt C., Hanschkow M., Popkova Y., Krause K., Klöting N., Blüher M., Schiller J., Heiker J.T. (2023). Differential expression of immunoregulatory cytokines in adipose tissue and liver in response to high fat and high sugar diets in female mice. Front. Nutr..

[83] Wu J.X., He Q., Zhou Y., Xu J.Y., Zhang Z., Chen C.L., Wu Y.-H., Chen Y., Qin L.-Q., Li Y.-H. (2023). Protective effect and mechanism of lactoferrin combined with hypoxia against high-fat diet induced obesity and non-alcoholic fatty liver disease in mice. Int. J. Biol. Macromol..

[84] Li C., Nie S.P., Zhu K.X., Ding Q., Li C., Xiong T., Xie M.Y. (2014). Lactobacillus plantarum NCU116 improves liver function, oxidative stress and lipid metabolism in rats with high fat diet induced non-alcoholic fatty liver disease. Food Funct..

[85] Duan M., Wang Y., Zhang Q., Zou R., Guo M., Zheng H. (2021). Characteristics of gut microbiota in people with obesity. PLoS ONE.

[86] Tilg H., Moschen A.R. (2024). Gut microbiome, obesity, and metabolic syndrome. Metabolic Syndrome: A Comprehensive Textbook.

[87] Santos-Marcos J.A., Perez-Jimenez F., Camargo A. (2019). The role of diet and intestinal microbiota in the development of metabolic syndrome. J. Nutr. Biochem..

[88] Kusnadi Y., Saleh M.I., Ali Z., Hermansyah H., Murti K., Hafy Z., Yuristo N.E. (2023). Firmicutes/Bacteroidetes Ratio of Gut Microbiota and Its Relationships with Clinical Parameters of Type 2 Diabetes Mellitus: A Systematic Review. Maced. J. Med. Sci..

[89] Zhou H., Liu K., Liu W., Wu M., Wang Y., Lv Y., Meng H. (2023). Diets enriched in sugar, refined, or whole grain differentially influence plasma cholesterol concentrations and cholesterol metabolism pathways with concurrent changes in bile acid profile and gut microbiota composition in ApoE-/-Mice. J. Agric. Food Chem..

[90] Magne F., Gotteland M., Gauthier L., Zazueta A., Pesoa S., Navarrete P., Balamurugan R. (2020). The Firmicutes/Bacteroidetes ratio: A relevant marker of gut dysbiosis in obese patients?. Nutrients.

[91] Crovesy L., Masterson D., Rosado E.L. (2020). Profile of the gut microbiota of adults with obesity: A systematic review. Eur. J. Clin. Nutr..

[92] Salazar-Jaramillo L., de la Cuesta-Zuluaga J., Chica L.A., Cadavid M., Ley R.E., Reyes A., Escobar J.S. (2024). Gut microbiome diversity within *Clostridia* is negatively associated with human obesity. mSystems.

[93] Chen R., Xu Y., Wu P., Zhou H., Lasanajak Y., Fang Y., Tang L., Ye L., Li X., Cai Z. (2019). Transplantation of fecal microbiota rich in short chain fatty acids and butyric acid treat cerebral ischemic stroke by regulating gut microbiota. Pharmacol. Res..

[94] Gao R., Zhu C., Li H., Yin M., Pan C., Huang L., Kong C., Wang X., Zhang Y., Qu S. (2018). Dysbiosis signatures of gut microbiota along the sequence from healthy, young patients to those with overweight and obesity. Obesity.

[95] Serena C., Ceperuelo-Mallafré V., Keiran N., Queipo-Ortuño M.I., Bernal R., Gomez-Huelgas R., Urpi-Sarda M., Sabater M., Pérez-Brocal V., Andrés-Lacueva C. (2018). Elevated circulating levels of succinate in human obesity are linked to specific gut microbiota. ISME J..

[96] Garcia-Mazcorro J.F., Mills D.A., Murphy K., Noratto G. (2018). Effect of barley supplementation on the fecal microbiota, caecal biochemistry, and key biomarkers of obesity and inflammation in obese db/db mice. Eur. J. Nutr..

[97] Larsen J.M. (2017). The immune response to *Prevotella* bacteria in chronic inflammatory disease. Immunology.

[98] Companys J., Gosalbes M.J., Pla-Pagà L., Calderón-Pérez L., Llauradó E., Pedret A., Valls R.M., Jiménez-Hernández N., Sandoval-Ramirez B.A., Del Bas J.M. (2021). Gut Microbiota Profile and Its Association with Clinical Variables and Dietary Intake in Overweight/Obese and Lean Subjects: A Cross-Sectional Study. Nutrients.

[99] Alili R., Belda E., Fabre O., Pelloux V., Giordano N., Legrand R., Bel Lassen P., Swartz T.D., Zucker J.-D., Clément K. (2022). Characterization of the Gut Microbiota in Individuals with Overweight or Obesity during a Real-World Weight Loss Dietary Program: A Focus on the Bacteroides 2 Enterotype. Biomedicines.

[100] Rios-Covian D., Arboleya S., Hernandez-Barranco A.M., Alvarez-Buylla J.R., Ruas-Madiedo P., Gueimonde M., de los Reyes-Gavilan C.G. (2013). Interactions between Bifidobacterium and Bacteroides species in co-fermentations are affected by carbon sources, including exopolysaccharides produced by bifidobacteria. Appl. Environ. Microbiol..

[101] Cheng J., Hu J., Geng F., Nie S. (2022). Bacteroides utilization for dietary polysaccharides and their beneficial effects on gut health. Food Sci. Hum. Wellness.

[102] Tamanai-Shacoori Z., Smida I., Bousarghin L., Loreal O., Meuric V., Fong S.B., Bonnaure-Mallet M., Jolivet-Gougeon A. (2017). *Roseburia* spp.: A marker of health?. Future Microbiol..

[103] Leite G., Barlow G.M., Rashid M., Hosseini A., Cohrs D., Parodi G., Morales W., Weitsman S., Rezaie A., Pimentel M. (2024). Characterization of the Small Bowel Microbiome Reveals Different Profiles in Human Subjects who are Overweight or have Obesity. Am. J. Gastroenterol..

[104] Tian X.Y., Xing J.W., Zheng Q.Q., Gao P.F. (2021). 919 syrup alleviates postpartum depression by modulating the structure and metabolism of gut microbes and affecting the function of the hippocampal GABA/glutamate system. Front. Cell. Infect. Microbiol..

[105] Franke T., Deppenmeier U. (2018). Physiology and central carbon metabolism of the gut bacterium *Prevotella copri*. Mol. Microbiol..

[106] Muscogiuri G., Cantone E., Cassarano S., Tuccinardi D., Barrea L., Savastano S., Colao A. (2019). Gut microbiota: A new path to treat obesity. Int. J. Obes. Suppl..

[107] Ishioh M., Nozu T., Okumura T. (2024). Brain Neuropeptides, Neuroinflammation, and Irritable Bowel Syndrome. Digestion.

[108] Lainez N.M., Jonak C.R., Nair M.G., Ethell I.M., Wilson E.H., Carson M.J., Coss D. (2018). Diet-induced obesity elicits macrophage infiltration and reduction in spine density in the hypothalami of male but not female mice. Front. Immunol..

[109] Park J.H., Yoo Y., Han J., Park Y.J. (2019). Altered expression of inflammation-associated genes in the hypothalamus of obesity mouse models. Nutr. Res..

[110] Szegletes T., Mallender W.D., Thomas P.J., Rosenberry T.L. (1999). Substrate binding to the peripheral site of acetylcholinesterase initiates enzymatic catalysis. Substrate inhibition arises as a secondary effect. Biochemistry.

[111] Kaizer R.R., da Silva A.C., Morsch V.M., Corrêa M.C., Schetinger M.R. (2004). Diet-induced changes in AChE activity after long-term exposure. Neurochem. Res..

[112] Saiyasit N., Chunchai T., Prus D., Suparan K., Pittayapong P., Apaijai N., Pratchayasakul W., Sripetchwandee J., Chattipakorn N., Chattipakorn S.C. (2020). Gut dysbiosis develops before metabolic disturbance and cognitive decline in high-fat diet-induced obese condition. Nutrition.

[113] Stilling R.M., van de Wouw M., Clarke G., Stanton C., Dinan T.G., Cryan J.F. (2016). The neuropharmacology of butyrate: The bread and butter of the microbiota-gut-brain axis?. Neurochem. Int..

[114] Bruce-Keller A.J., Salbaum J.M., Luo M., Blanchard E.T., Taylor C.M., Welsh D.A., Berthoud H.R. (2015). Obese-type gut microbiota induce neurobehavioral changes in the absence of obesity. Biol. Psychiatry.

[115] Gates N.J., Vernooij R.W., Di Nisio M., Karim S., March E., Martinez G., Rutjes A.W. (2019). Computerised cognitive training for preventing dementia in people with mild cognitive impairment. Cochrane Database Syst. Rev..

[116] Trecroci A., Cavaggioni L., Rossi A., Moriondo A., Merati G., Nobari H., Ardigò L.P., Formenti D. (2022). Effects of speed, agility and quickness training programme on cognitive and physical performance in preadolescent soccer players. PLoS ONE.

[117] Sun X., Li Y., Cai L., Wang Y. (2021). Effects of physical activity interventions on cognitive performance of overweight or obese children and adolescents: A systematic review and meta-analysis. Pediatr. Res..

[118] Tait J.L., Collyer T.A., Gall S.L., Venn A.J., Dwyer T., Fraser B.J., Moran C., Srikanth V.K., Callisaya M.L. (2024). Associations of midlife fitness and obesity profiles with cognitive function. Eur. J. Sport Sci..

